# [Retracted] MicroRNA‑520a attenuates proliferation of Raji cells through inhibition of AKT1/NF‑κB and PERK/eIF2α signaling pathway

**DOI:** 10.3892/or.2024.8706

**Published:** 2024-01-22

**Authors:** Xiaojuan Wang, Pei Wang, Yan Zhu, Zhi Zhang, Jinqian Zhang, Hongwei Wang

Oncol Rep 36: 1702–1708, 2016; DOI: 10.3892/or.2016.4975

Following the publication of this paper, it was drawn to the Editors' attention by a concerned reader that a pair of the lanes featured in the western blot gel for Fig. 3B on p. 1705 were strikingly similar, suggesting that the same data may have been imported into the figure to represent data that were intended to show the results from differently performed experiments. In addition, the western data shown in Fig. 6 on p. 1706 for the GRP78 and the p-PERK proteins also appeared to be strikingly similar, such that the same data may have also been inserted into this figure to show the results from different experiments. Finally, certain of the data in this paper were shown to have appeared in a previous publication that featured some of the same authors, albeit in different form [Zhang L, Huang D, Wang Q, Shen D, Wang Y, Chen B, Zhang J and Gai L: MiR-132 inhibits expression of SIRT1 and induces pro-inflammatory processes of vascular endothelial inflammation through blockade of the SREBP-1c metabolic pathway. Cardiovasc Drugs Ther 8: 303–311, 2014].

Owing to the fact that some of the contentious data in the above article had already been published elsewhere prior to its submission to *Oncology Reports*, and because of an overall lack of confidence in the presented data, the Editor has decided that this paper should be retracted from the Journal. The authors were asked for an explanation to account for these concerns, but the Editorial Office did not receive a reply. The Editor apologizes to the readership for any inconvenience caused.

